# Sleep Abnormalities and Risk of Alzheimer’s Disease

**DOI:** 10.1007/s11910-025-01451-5

**Published:** 2025-10-13

**Authors:** Merve Aktan Süzgün, Qi Tang, Ambra Stefani

**Affiliations:** Department of Neurology, Medical University Innsbruck, Anichstraße 35, Innsbruck, 6020 Austria

**Keywords:** AD, Cognitive decline, Sleep architecture, Circadian rhythm, Sleep disorders

## Abstract

**Purpose of Review:**

This review aimed at investigating sleep abnormalities as risk factors for Alzheimer’s disease (AD), with a focus on their potential utility in early disease detection and risk modification.

**Recent Findings:**

Impaired sleep quality, circadian misalignment, and disruptions in sleep architecture are significantly associated with an elevated risk of AD. Moreover, excessive or insufficient sleep, reductions in slow-wave and REM sleep, and fragmented rest-activity rhythms have been linked to early alterations in amyloid-β and tau biomarkers, even in cognitively unimpaired individuals. Various sleep disorders have also been identified as independent contributors to AD risk, particularly among genetically susceptible populations.

**Summary:**

Sleep and circadian disturbances, as well as changes in sleep architecture, represent easily detectable and modifiable risk factors for Alzheimer’s disease. Integrating sleep and sleep-based metrics into preventive strategies may enhance early identification and offer novel avenues for intervention, modulating the risk of Alzheimer’s disease.

## Introduction

Sleep plays a critical role in brain health, and growing evidence implicates sleep disturbances as key contributors to Alzheimer’s disease (AD) risk. Here we synthesize findings across multiple domains of sleep research to better understand this relationship, based mainly on recent literature from the past three years. We first focus on general sleep measures and their association with AD risk, including macro- and microstructural sleep parameters. We then explore how specific sleep disorders –insomnia, obstructive sleep apnea (OSA), central disorders of hypersomnolence (CDH), and restless legs syndrome (RLS)– may affect the risk of AD, discussing potential mechanisms. Together, these findings highlight sleep as an early marker of AD vulnerability and a modifiable risk factor.

## Sleep Measures and AD Risk

### Macrostructural Sleep Measures

#### Sleep Duration

The relationship between sleep duration and dementia continues to drive research [1]. Previous evidence suggested a nonlinear “U-shaped” relationship between self-reported sleep duration and AD risk, with both short (< 4 h) and long (> 8 h) sleep durations linked to increased risk [2, 3]. Abnormal sleep duration correlates also with structural brain changes. In a healthy population-based cohort, every additional hour of self-reported sleep above 7 h/day was associated with 0.10–0.25% smaller brain volumes [4]. However, recent findings indicate that the relationship between sleep duration and dementia risk may be more complex, with long-term high variability in sleep duration potentially contributing to cognitive decline in older adults [5]. A meta-analysis suggests that transitioning from short/moderate to long sleep duration is itself associated with accelerated cognitive decline [6]. This finding is in line with longitudinal studies showing that a consistent increase in time in bed (TIB) and prolonged 24-h sleep duration (≥ 9 h/day) can appear 12 years before the diagnosis of dementia [7], and that individuals with increased 24-hour sleepiness over 5 years have a doubled dementia risk (OR = 2.21) compared to those with stable sleep [8]. Thus, changes in sleep patterns over time may be key for early identification of subjects at risk of developing AD. Furthermore, ideal sleep duration vary throughout the life span, and maintaining proper sleep time is essential for cognitive function in ageing populations. In a 10-year cohort of adults ≥ 70 years, those with self-reported sleep duration > 8 h had a 2-fold risk of AD compared to those sleeping 7–8 h [9]. Short sleep duration or insufficient sleep appear to have an even greater impact on adults. Among individuals aged < 70 years, short sleep (< 7 h) was linked to higher dementia risk (HR = 1.81). Similarly, 50 and 60 year old adults reporting ≤ 6 h of sleep have higher dementia incidence (HR = 1.22 and 1.37, respectively) at 25-year follow-up [10]. These data suggest that specific sleep duration patterns in different age groups might help identify subjects at high risk for AD.

#### Circadian Rhythm

Substantial evidence supports a strong association between circadian rhythm disruption and AD. This relationship is bidirectional, complex, and interactive [11]. In animal models, disrupted sleep-wake cycles and dysregulated expression of clock genes have been shown to accelerate the aggregation and deposition of amyloid-β (Aβ) and tau phosphorylation. In humans, emerging evidence suggests that circadian rhythm disruption precedes the onset of dementia and likely contributes to AD pathogenesis. Recent studies suggest that 24-hour rhythm integrity, as measured by wearable devices, may serve as an early marker of neurodegenerative disease. A longitudinal actigraphy study in older adults found that reduced diurnal amplitude and lower 24-hour activity levels were associated with an increased AD risk and greater cognitive decline [12]. Also, several studies found that suppressed and fragmented 24-h rest-activity rhythms precede the onset of dementia or mild cognitive impairment (MCI), are linked to increased Aβ burden and phosphorylated-tau (p-tau) level, and may serve as risk biomarkers for preclinical dementia in middle-aged and older adults [13–17]. Neuroimaging studies linked greater 24-h rest-activity rhythm fragmentation and poorer synchronization to the 24-h light-dark cycle to reduced cortical thickness in frontal, temporal, parietal, and occipital regions in high-risk adults [18]. Despite earlier research reported that 24-hour activity rhythm fragmentation or stability does not affect the risk of dementia [19], abnormalities of rest-activity rhythms seem to be a potentially modifiable AD risk factor. Moreover, irregular sleep-wake timing among older adults, e.g. self-reported early (≤ 6am) and late (> 8am) rising time, was associated with lower hippocampal volume [20] and worse cognitive performance [21]. These alterations may indicate circadian rhythm disruptions or sleep disorders.

#### Sleep Deprivation

Sleep deprivation has been observed to increase Aβ deposition and tau phosphorylation in both animal models and clinical research. Studies in healthy individuals have reported that different kinds of sleep deprivation (e.g., one night or multiple consecutive nights, total or partial sleep deprivation) lead to increased Aβ burden in the hippocampus and thalamus, and to high levels of plasma or cerebrospinal fluid (CSF) biomarkers, including Aβ38, Aβ40, Aβ42, p-tau and total tau [22–24]. A single night of sleep deprivation increased overnight CSF Aβ40 and Aβ42 levels by ~ 10% in middle-aged adults [25]. Acute sleep deprivation can also increase plasma total tau levels in healthy young adults [26] and may disrupt physiological processes modulating site-specific tau phosphorylation, leading to p-tau forms involved in early neurodegeneration [27]. Of note, one study found (∼35–55% increased Aβ40, Aβ42 and p-tau 181 concentration in CSF, and d ~ 5–15% decreased plasma concentrations during sleep deprivation [28]. The authors suggested that sleep deprivation decreased CSF-to-blood clearance. However, CSF and plasma Aβ42/40, p-tau 181/tau 181, and p-tau 181/Aβ42 ratios were not investigated, so that increased production cannot be excluded.

Other emerging potential AD biomarkers include circulating microRNAs (miRNAs) involved in sleep and circadian regulation. In a randomized controlled trial simulating shiftwork in healthy young men, one night of total sleep deprivation significantly increased plasma miR-127-3p and miR-142-3p levels in the morning compared to a full night sleep [29]. As miR-142-3p targets neuroinflammation pathways and genetic variations in miR-142 promoter have been associated with AD risk, the authors suggest a potential role of miR-142 in AD pathogenesis.

A previous meta-analysis of 71 population studies showed that consistent sleep deficit affected overall cognitive performance and especially sustained attention, and that the progressive neurocognitive decline accumulated over consecutive days was related to sleep loss severity [30]. Similarly, a recent randomized crossover study in healthy adults over two different 6-weeks-period sleep conditions found that consistent, adequate sleep (≥ 7 h/night) enhanced working memory and response inhibition, while a 1.5-hour nightly sleep reduction impaired expected cognitive gains [31].

#### Subjective and Objective Sleep Quality

A systematic review has showed that individuals reporting sleep disturbances are at higher risk of developing AD compared to those without such complaints [1]. Subjective sleep quality is commonly evaluated using questionnaires such as the Pittsburgh Sleep Quality Index (PSQI), whereas objective sleep quality is assessed by polysomnography (PSG) or through measures of nocturnal activity to infer nighttime wakefulness using actigraphy. Several studies have demonstrated that poor subjective sleep quality is associated with impaired cognitive function, subjective cognitive decline, and an increased risk of developing dementia [32–34]. Recent studies suggest that low sleep efficiency (SE), rather than total sleep time (TST), predicts the risk of AD [35, 36]. Sleep fragmentation metrics such as wakefulness after sleep onset (WASO), frequent long wake episodes and nocturnal activities were also linked with cognitive decline and higher risk of AD [36, 37]. These findings echo earlier studies demonstrating that poor nocturnal sleep quality (longer TIB, prolonged sleep latency, increased WASO and reduced SE) increases risk of AD [19], whereas compensatory daytime napping has been associated with a reduced risk [38]. In line with these findings, healthy older adults with higher sleep continuity and reduced nocturnal restlessness tend to perform better on memory tasks [39], suggesting that sleep fragmentation and night-time wakefulness reduce the beneficial effects of sleep against neurodegeneration and increase AD risk [40]. In addition to cognitive changes, increased sleep fragmentation and subjective poor sleep quality have been linked to Aβ and tau burden in non-demented populations [41–43], as well as to elevated plasma neurofilament light chain (NfL) levels [34]. Nevertheless, direct causal evidence for these self-reported and actigraphy-measured sleep parameters remains limited [38].

#### Sleep Stages and Sleep Latency

Slow-wave sleep (SWS) plays an essential role in memory consolidation and brain health [44]. Loss of SWS is recognized as a risk factor for dementia [45–47]. Although SWS proportion physiologically declines with age [48, 49]. greater reductions in SWS percentage over time, rather than cross-sectional individual differences, seem to be more important for determining dementia risk, and age-related SWS decline is accelerated in APOE-ε4 carriers [45]. Moreover, a correlation between reduced REM sleep and cognitive impairment was reported in patients with AD [50]. Although REM sleep disturbances are consistently linked to Parkinson’s disease and other neurodegenerative disorders, recent studies have turned their focus to the role of REM sleep dysfunction in cognitive decline and AD [39, 51–53]. During PSG, both lower REM sleep percentage and duration have been observed in APOE-ε4 carriers without cognitive impairments [53]. Healthy older adults with lower proportions of REM sleep at baseline were more likely to develop later on atrophy in the inferior parietal region, a brain region commonly affected in the early stages of AD [46]. Another study reported an association between prolonged REM sleep latency and increased Aβ plaque deposition and elevated plasma p-tau-18 levels, independent from cognition and other objective sleep parameters [54]. Thus, REM sleep alterations may contribute to AD progression or could be an early sign of neurodegeneration.

### Microstructural Sleep Measures

Brain activity during different sleep stages, such as slow wave activity (SWA), sleep spindles and other sleep oscillations, is associated with cognition and memory. Numerous studies have shown alterations in sleep microstructure and electroencephalography (EEG) spectral power in patients with AD [55–57]. However, it is not clear whether these alterations precede the diagnosis of AD or could accelerate its onset. A recent systematic review found that changes in sleep microstructural parameters were associated with cognitive decline and suggested that those changes may precede cognitive impairments in preclinical and early AD [36]. Previous evidence in cognitively normal middle-aged/older adults showed that altered sleep microstructure is associated with increased AD biomarkers. For example, decreased proportion of SWA and reduced sleep spindles density were related to cerebral Aβ accumulation and tau-related early dysfunction, and could predict late-life Aβ and tau burden [35, 58]. Conversely, a high proportion of SWA could prevent memory impairment in individuals with high Aβ burden [59]. Animal models further confirmed that slow-wave impairments arise prior to amyloid plaque deposition [60, 61]. However, another recent study found that slow delta power positively correlated with gray matter volume in fronto-cingular regions, rather than Aβ accumulation [62]. This inconsistency may be related to differences in follow-up time, underlying the need for further longitudinal studies up to several years to better clarify the relationship between delta power and amyloid pathology. Moreover, abnormality and imprecision of the coupling events, such as slow wave–spindle coupling and slow wave–theta burst coupling, correlated with changes in CSF biomarkers and contributed to poorer cognitive trajectories in ageing [63, 64]. Among individuals who subsequently develop MCI or dementia, increases in sigma, alpha, and theta waves absolute power have been observed to precede clinical diagnosis by at least five years [65]. Furthermore, abnormalities in fast-to-slow spectral power ratio (e.g. abnormal beta/theta ratio) and REM-EEG slowing show promise as biomarkers to distinguish MCI from healthy ageing [66, 67].

### Mechanistic Insights into Sleep Alterations and AD Pathology

**Fig. 1:**
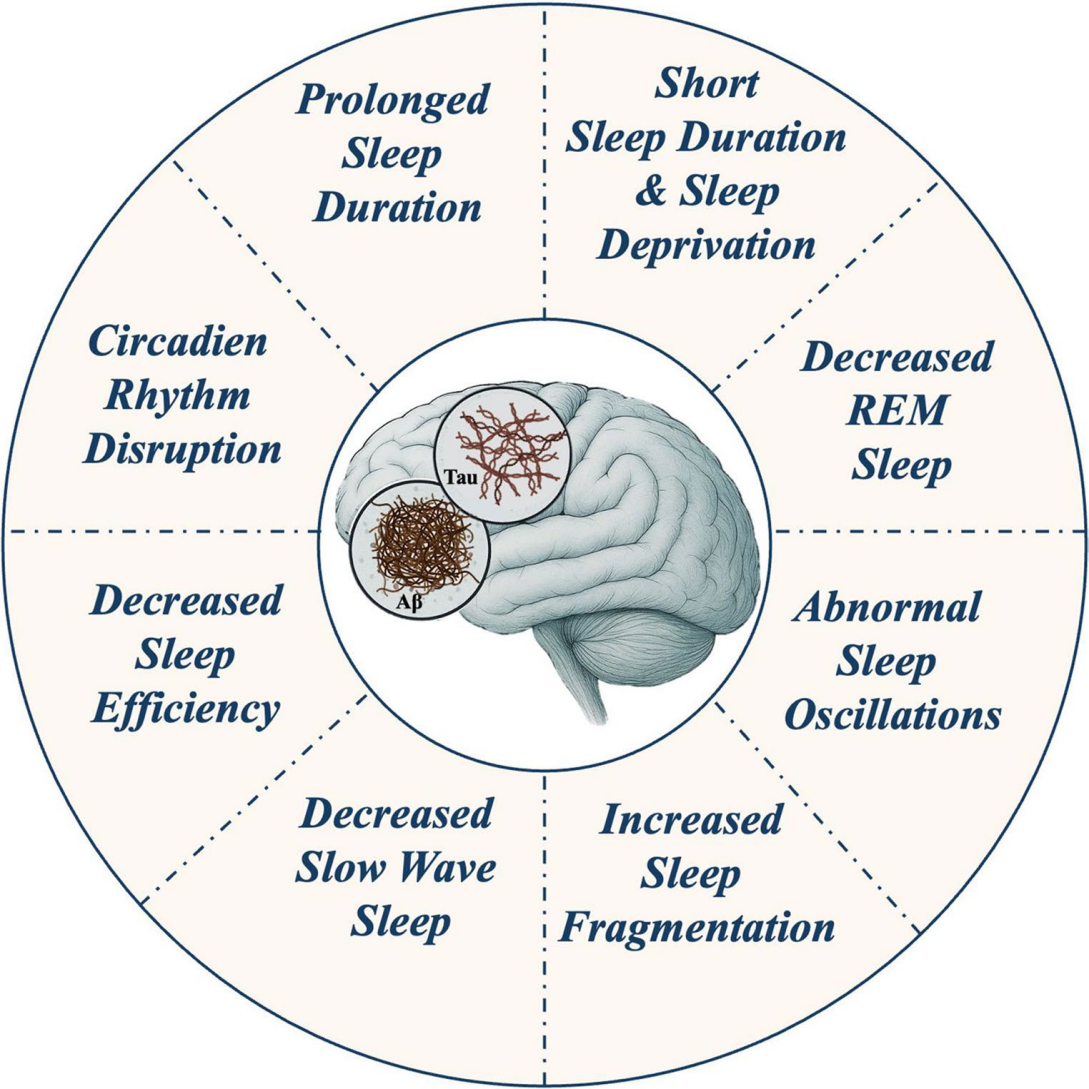
Sleep abnormalities reported to be associated with an increased risk of Alzheimer’s disease

Emerging evidence suggests a bidirectional relationship between sleep and AD [68]. Animal models and clinical research based on cognitive, structural imaging and fluid biomarkers well document a negative effect on AD risk of sleep patterns’ disruptions, alterations in SWS and REM sleep, poor sleep quality, and circadian rhythm disturbances (Fig. 1). Both insufficient and prolonged sleep durations, as well as sleep fragmentation may contribute to cognitive decline over time. Although extensive research has established the presence of both macroscopic and microstructural alterations in sleep and in circadian rhythms preceding the onset of AD, the underlying mechanism driving these changes and their contribution to AD onset and cognitive decline remain unclear.

The glymphatic system is a potential mediator of the relationship between sleep disturbance and AD pathophysiology [69]. This glial-dependent waste clearance system of the brain utilizes the cerebral perivascular space formed by the astrocytic end-feet, which express aquaporin-4 water channels (AQP4). and facilitates long-distance circulation and brain waste clearance through CSF oscillations, particularly during SWA in NREM sleep. A genetic variant in the AQP4 gene (specific single nucleotide polymorphisms), has been linked to reduced sleep quality and an increased Aβ burden [70]. Sleep disturbances and circadian dysregulation likely hinder glymphatic flow and contribute to amyloid accumulation and tau deposition through altered polarization of AQP4 channels in the perivascular astrocytic end-feet. Further longitudinal and mechanistic studies are needed to better clarify the interplay between sleep abnormalities, glymphatic system, and AD progression.

## Risk of AD in Insomnia Disorder

### Prevalence and Cognitive Measures

Approximately 15% of AD cases can be attributed to sleep problems [71]. Insomnia, as the most common sleep disorder, may contribute to a bidirectional cycle of cognitive decline and poor sleep, leading to impairments in attention, memory, and executive function [72–74]. Individuals with sleep-onset insomnia tend to exhibit more cognitive symptoms compared to those with maintenance insomnia [75]. A recent meta‑analysis showed that insomnia can cause a roughly 1.5‑fold increased risk of developing AD compared to normal sleepers [76].

### Biofluids and Neuroimaging Measures

In addition to measurable cognitive deficits, insomnia in cognitively unimpaired adults is associated with alterations in AD biomarkers and neuroanatomical integrity. CSF Aβ42 in patients with chronic insomnia was significantly increased and correlated with poor sleep quality as assessed by PSQI scores [77]. Neuroimaging studies revealed a widespread reduction in cortical and subcortical gray matters among insomnia individuals in the prefrontal cortex, temporal lobe, bilateral precuneus, posterior cingulate cortex, thalamus and hippocampus, together with decreased white matter diffusivity [74, 78]. These findings underscore the link between insomnia and AD‑related neurodegenerative processes.

### Treatment of Insomnia Disorder

#### Benzodiazepines – Possible Risk Factor for AD

Despite being recommended only for short-term treatment [79], benzodiazepine (BZD) and related Z-drugs are commonly used for the treatment of insomnia disorder. While some studies have reported only a weak association between BZD/Z-drugs and dementia [80, 81], growing evidence suggests a significant increase in the risk of AD and all-cause dementia among older adults using BZD/Z-drugs compared to non-users [82]. For example, one study demonstrated a 43–51% higher risk of AD among older adults who had used BZD within the past five years [83] and a meta-analysis reported a 21% increased risk of AD among older adults who were BZD users. Zolpidem, a commonly used Z-drug, was associated with a 28% greater risk of developing AD and dementia relative to non-users [84]. A relationship between BZD and increased incidence of MCI in cognitively normal older adults has been shown as well, independently from APOE-ε4 status [85]. Thus, BZD/Z-drugs may represent a modifiable risk factor for both MCI and AD.

The influence of different types of BZD/Z-drugs on dementia risk remains controversial. Several studies found no significant difference in dementia risk when comparing different subcategories of BZD/Z-drugs, like short/medium-acting or long-acting BZD/Z-drugs [76, 78], or conflicting results, as outlined below. However, the duration and intensity of BZD/Z-drugs exposure seem to play a role [86]. One study found that the risk of AD increased with exposure density and was more pronounced among long-acting BZD users [83]. Conversely, another study reported that only short half-life BZD/Z-drugs were associated with an elevated dementia risk at high doses, especially in female patients, indicating potential sex-specific heterogeneity [87]. Of note, a recent retrospective cohort study reported an inverse relationship between sedatives use and the incidence of AD and found that Z-drugs were associated with a greater risk reduction of AD than BZDs [88]. This apparent paradox may be due to the dual role of GABAergic modulation in AD [84], i.e. misfolded Aβ impairs the function of GABAergic neurons and decreases GABA neurotransmitter levels, leading to dysfunction of synaptic activity, neural network and ultimately cognitive dysfunction. Drugs targeting GABA-receptors stimulate the GABA system, and in AD mice models led to a decrease in Aβ production and cognitive improvements, showing neuroprotective functions. However, prolonged use of GABA agonists may diminish endogenous GABA-receptor activity and impair the brain’s capacity to recruit alternative neural networks. Further, a study in mice revealed that BZD use induced microglial activation by BZD binding to the surface translocator proteins, which impaired the structural plasticity of dendritic spines and caused cognitive decline [89]. Taken together, long‑term risks and benefits of BZD and Z-drugs use must be carefully weighted, in particular among older adults, and CBTI recommendation should be the first-line treatment for insomnia, as recommended by international guidelines (79).

#### Dual Orexin Receptor Antagonists (Doras) – Potential Neuroprotective Role

Dual Orexin Receptor Antagonists (DORAs), namely lemborexant, daridorexant, and suvorexant, recently approved for insomnia treatment, may decrease the risk of AD [90]. DORAs have been reported to reduce Aβ deposition and improve synaptic plasticity, thus suggesting neuroprotective effects [91]. Suvorexant has been shown to acutely decrease tau phosphorylation and Aβ concentrations in the central nervous system [92]. Thus, DORAs are a promising treatment of insomnia with a favorable risks/benefits balance in older adults and in patients at risk of AD. Yet, their longitudinal effects on AD pathophysiology need further investigation.

## Risk of AD in Obstructive Sleep Apnea

OSA is the most common form of sleep-related breathing disorders (SRBD) with a prevalence of 22% (range 9–37%) in men and 17% (range 4–50%) in women [93], affecting a large portion of middle-aged and older adults and posing a risk of cognitive decline and AD. Other forms of SRBD, like central apnea or hypoventilation syndromes, will not be discussed here due to their relatively low prevalence and limited data about AD risk.

### Prevalence Measures

The frequent co-occurrence of AD and OSA (up to 89%, with a male dominance) [94, 95] raised suspicion about a causative role of OSA in AD pathophysiology. Indeed, large cohort studies showed an increased risk of cognitive impairment and AD in patients with OSA. In a US cohort with 19,017 participants, those with OSA had 1.6 times more cognitive impairment compared to those without, independent from demographics, comorbidities and lifestyle [96]. A meta-analysis with 1,333,424 OSA patients documented a 1.4-fold increased risk of developing neurocognitive disorder and a 1.2 times increased risk for AD based on ICD diagnoses [97]. However, some studies revealed an increased risk of all-cause dementia in OSA [98], but no specific AD risk [99]. In two large genome-wide-association studies (GWAS) using mendelian randomization analysis including OSA (*n* = 523,366 − 16,761) and AD (*n* = 94,437 − 71,880), OSA was not causally associated with AD risk [100, 101]. These inconsistent reports might be related to different genetic susceptibility of investigated populations, since it was shown in APOE-ε4 carriers that the presence of OSA and high apnea-hypopnea index (AHI) was associated with a faster cognitive decline and worse cognitive outcomes [102–104]. Moreover, in patients with MCI, a high OSA polygenic risk score was correlated with MCI-to-AD progression, higher Aβ deposition and greater cognitive decline [105]. Thus, the presence of genetic risk for either AD or OSA seems to affect bi-directionally the prevalence of the other disease [106].

### Cognitive Measures

The cognitive domains predominantly affected in patients with OSA are attention, working memory, psychomotor speed, vigilance, and executive functions, with a mainly subcortical dementia pattern, possibly secondary to microvascular damage [107]. Despite the increased risk of AD, OSA is less associated with an amnestic syndrome with predominant memory deficits [107, 108]. Of note, in 81 adults with OSA AHI, respiratory disturbance index and oxygen desaturation index (ODI) during REM sleep and their REM/NREM ratio were negatively associated with total learning and long-delay recall, with a stronger association in APOE-ε4 carriers [109]. In line with these findings, Tan et al. [110] found an association between REM-AHI ≥ 5/hour and worse Trail-Making-Test performance in older adults. This suggests that OSA during REM sleep may particularly impact cognitive function.

### Blood and CSF Measures

In line with the studies discussed above, there is evidence of altered blood and CSF Aß and tau levels in patients with OSA [111]. A recent meta-analysis showed significantly higher CSF Aß40 and blood total Aß, Aß40, Aß42 and total-tau in patients with OSA compared to controls, independent from age, body-mass-index (BMI) and AHI; however, no difference in blood p-tau and CSF Aß42, p-tau and total-tau was found [112]. Another meta-analysis revealed significantly higher plasma total- and p-tau levels in patients with OSA compared to controls [113]. Nevertheless, some studies reported lower CSF Aβ40 and Aβ42, higher CSF tau/Aβ42 ratio, and unchanged CSF tau in OSA compared to controls, which were attributed to different preclinical stages of AD [111, 114]. In cognitively unimpaired older adults, OSA severity correlated with the annual decrease in CSF Aβ42 during two-year follow-up, predominantly in APOE-ε4 carriers [115]. Studies grouping OSA patients as at low- and high-risk of AD based on plasma total-tau and Aß42 found that OSA patients with high AD risk had higher SRBD indices, lower oxygen saturation, increased arousal responses, and shorter SWS duration compared to those with low AD risk [116, 117]. Besides changes in amyloid and tau, there are also findings on increased levels of inflammation, oxidative stress and lipoprotein markers in OSA, similar to those reported in AD [111].

### Neuroimaging Measures

Structural and functional imaging play a substantial role in disentangling the association between cognitive outcomes and different biomarkers. Structural imaging changes in OSA reflect a pattern of AD-related neurodegeneration with reduced cortical thickness in the bilateral temporal lobes [118] and less specific signs of neurodegeneration, like excessive iron accumulation in the superior frontal, orbital, angular, supramarginal, and middle temporal gyri [119]. Interestingly, in both studies abnormal MRI patterns, rather than sleep fragmentation or changes in SWS, correlated with nocturnal hypoxemia [118, 119]. In a FDG-PET study, significantly reduced glucose consumption was reported in the bilateral precuneus, posterior cingulate cortex, and frontal areas in patients with untreated OSA compared to controls; increased plasma total- and p-tau levels correlated with these changes [120]. Similarly, a resting-state fMRI study in OSA patients found that higher AHI correlated with lower functional connectivity between (i) the medial prefrontal cortex and bilateral hippocampi, and (ii) the left hippocampus and both posterior cingulate cortex and precuneus [121]. Using amyloid-PET, Cavuoto et al. reported an association between Aβ burden and nocturnal hypoxemia, and a negative correlation between Aβ burden and executive function independent from other sleep parameters [122].

### OSA Treatment

The above-mentioned changes might be reversible with successful treatment of OSA. Kong et al. investigated an OSA cohort with high baseline plasma Aβ40, total- and p-tau levels, who underwent uvulopalatopharyngoplasty [123]. Sixth-month after surgery, fluid biomarkers were significantly lower and the MoCA score was higher than at baseline; the changes in fluid biomarkers were correlated with the change in cognition scores [123]. Liguori et al. investigated the effect of PAP treatment on the progression of cognitive impairment in patients with MCI and comorbid OSA [124]. After 1-year follow-up, MCI patients with good PAP adherence (mean use ≥ 4 h per night for > 5 nights/week with residual AHI < 5/h) showed a milder progression in clinical dementia rating scale compared to MCI patients not adherent to the PAP therapy [124]. Fernandes et al. also documented an improvement in the attention domain and a global increase in 18 F-FDG uptake after 1 year of PAP therapy [120]. A recent meta-analysis with 60,840 OSA patients confirmed the positive effect of PAP with reduced MCI/AD incidence, later MCI onset, and better executive functions under treatment [125, 126].

### Possible Pathophysiological Mechanisms

**Fig. 2:**
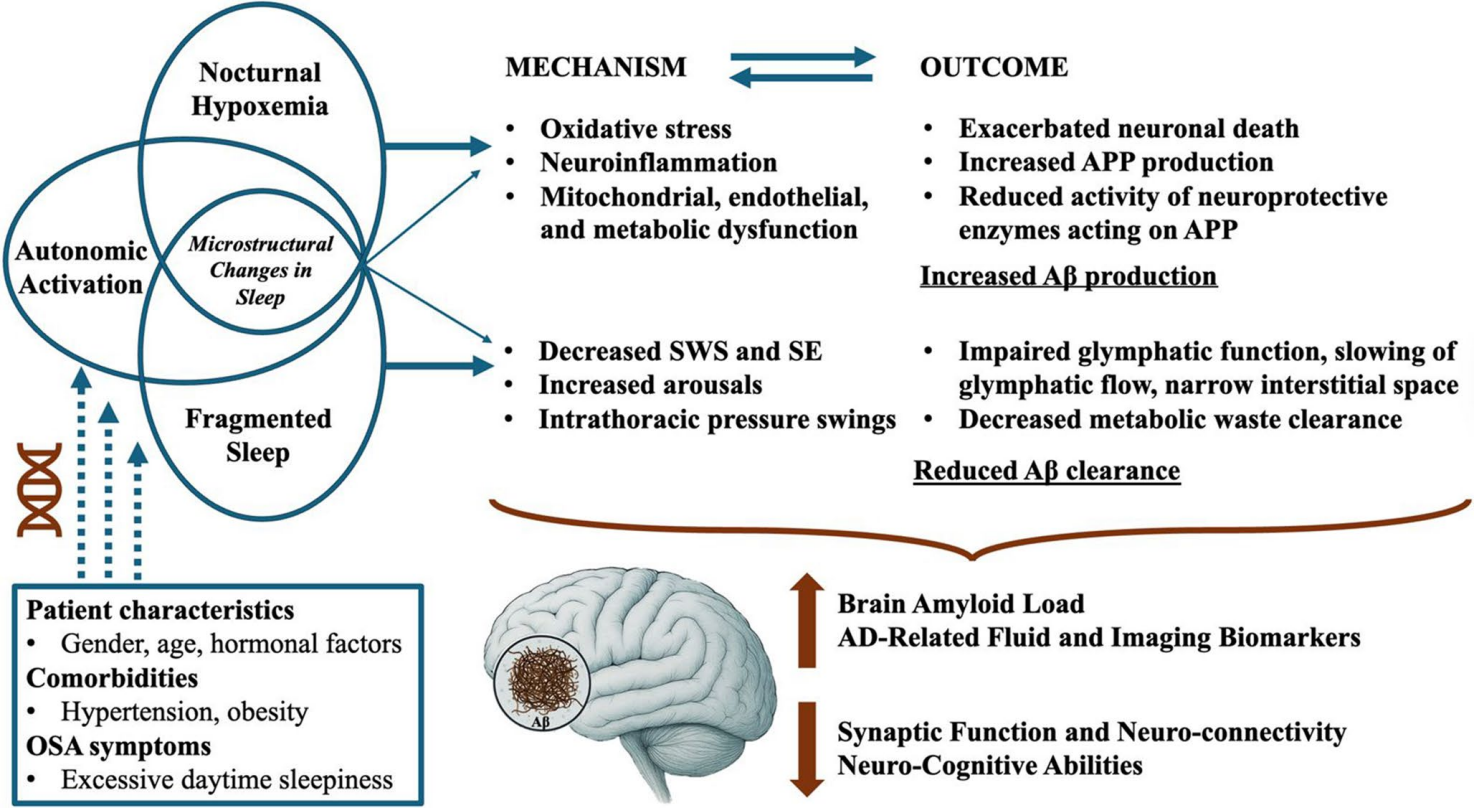
Proposed mechanisms increasing brain Aβ accumulation in patients with obstructive sleep apnea either via increased production or via reduced clearance of Aβ Legend: *Aβ: Amyloid beta*,* APP: Amyloid precursor protein*,* OSA: Obstructive sleep apnea*,* SE: Sleep efficiency*,* SWS: Slow-wave sleep*

In Fig. 2, we provide an overview of potential pathways linking OSA to an increased risk of AD. Apart from the already-mentioned genetic susceptibilities and demographic/comorbid factors, basic and clinical research agree on two main mechanisms that trigger increased Aβ accumulation via either increased production or decreased degradation of abnormally folded proteins. The first mechanism is primarily driven by intermittent nocturnal hypoxemia (as measured by AHI, ODI or percent of time spent with low levels of oxygen saturation) and to a lesser extent by decreased SWS and increased sympathetic tonus during sleep [113, 127]. Through oxidative stress, neuroinflammation, mitochondrial dysfunction and various metabolic disturbances, intermittent nocturnal hypoxemia leads to increased neuronal death and, as a result, increased production of Aβ with reduced activity of neuroprotective enzymes acting on amyloid precursor proteins [111]. The second mechanism is explained by impairment of the glymphatic function due to decreased SWS, frequent arousals with autonomic activation and increased intrathoracic pressure, impairing Aβ clearance [111]. Besides direct effects on Aβ production and clearance dynamics, both pathways cause substantial changes in sleep structure, which have been reported to be associated with impaired cognitive ability. Those include decreased SE, increased WASO, fragmented sleep, higher arousability [127], reduced spindle density, power and frequency, reduced percentage of fast spindles [128], decreased SWA during NREM sleep, altered K-complex density [127], increased A3 and reduced A1 and A2 indices in cyclic-alternating-pattern [129], low normalized EEG power [130], greater REM-EEG slowing [131] and altered Odds-ratio-product [130].

So far, the differential negative effect of sleep fragmentation versus nocturnal hypoxemia on AD pathology has been mainly investigated in AD animal models. After exposure to chronic intermittent hypoxia, APP/PS1 mice –AD model with mutated human amyloid precursor protein and presenilin 1 genes– showed spatial learning and memory deficits along with hippocampal long-term potentiation dysfunction [132], whereas transient intermittent hypoxia was found to be involved in both Aβ overproduction and tau phosphorylation without behavioral changes [133]. On the other hand, there are contradictory findings about chronic sleep fragmentation, which has been reported to affect either the tau or the amyloid system [134, 135]. In this regard, animal research needs to be cautiously evaluated, since the experimental SWS deprivation might be too strict and deep and not comparable to physiological human sleep. Patient characteristics, comorbidities, or OSA symptoms’ profile might also play a role in the pathophysiological outcomes, so that fine-tuned phenotyping of OSA patients might help better understanding these pathophysiological mechanisms.

## Risk of AD in Central Disorders of Hypersomnolence

CDH (e.g. narcolepsy, idiopathic hypersomnia, insufficient sleep syndrome) are a group of sleep disorders characterized by excessive daytime sleepiness (EDS) and/or hypersomnia mainly affecting the young or middle-aged population. EDS and/or hypersomnia might either be a risk factor for AD development or an early sign of ongoing AD pathophysiology. Here we will focus only on EDS/hypersomnolence as a risk factor for AD.

### Cognitive Disturbance and AD in Patients with EDS/Hypersomnia

Increased cognitive disturbances and increased risk of all-cause dementia and, specifically, of AD in patients with EDS/hypersomnia were mainly revealed by large population cohorts. A recent study with 2,187,089 European participants from five different GWAS datasets showed a negative correlation between EDS and average cortical thickness, and of daytime napping with fluid intelligence and hippocampal volume [136]. In line with this, 1,778 participants who reported EDS performed significantly worse than controls on the cognitive composite score, after controlling for covariates [137]. A recent meta-analysis of 31 studies documented a trend toward a higher risk for AD (1.2 times) in patients with daytime napping/EDS [2]. In addition, AD risk was 1.6 times higher in patients with long sleep time (> 8 h) compared to those with normal sleep duration (5–8 h) [2]. These findings are supported by amyloid-PET imaging data, showing an association between baseline EDS and regional Aβ accumulation in the anterior and posterior cingulate, precuneus, and parietal regions in older adults without dementia [138]. However, there is also contradictory evidence that subjects with EDS are at a higher risk of all-cause dementia (HR 1.3) and vascular dementia (HR 2.1), but not specifically of AD (HR 1.1) [139]. This heterogeneity might be attributed to the fact that EDS has significant associations with race, ethnicity, APOE-ε4 status, history of cardiovascular disease, and presence of depression, which need to be taken into account as confounders [137]. As an example of racial differences, a recent cross-sectional and longitudinal study in an African American cohort investigating the associations of self-report sleep duration (SRSD) and EDS with plasma Aβ level and cognition showed that greater sleepiness was related to poorer overall levels of word list learning [140]. However, neither SRSD nor EDS was associated with cross-sectional or longitudinal Aβ level [140]. More comprehensive cohort studies assessing possible confounders are needed to clarify the link between EDS/hypersomnia an AD risk.

### EDS/Hypersomnia and Development of AD

In a longitudinal study on 1,749 cognitively unimpaired older adults with up to 14-years follow-up, assessed cognition assessments at baseline, 2, 8, 10, 12 and 14 years, and diagnosed 182 dementia cases [7]. Baseline sleep duration, especially ≥ 9 h, and becoming long sleeper over time were significantly associated with development of dementia [7].

Three possible pathological pathways have been proposed to explain the link between EDS/hypersomnia and AD:

#### Role of Neuro-Inflammation

In older adults with a parental history of AD and whose age was nearing their expected AD onset [141], the presence of hypersomnia positively correlated with CSF neuroinflammatory markers (IL-6, MCP-1, and global score) that were also higher in APOE-ε4 carriers [141]. A study with 260 cognitively unimpaired older adults revealed a positive association between ESS scores and CSF IL-6 and NfL levels [142]. In addition, in a subgroup of patients with already altered p-tau/Aβ42 ratio, suggestive of amyloid positivity, ESS scores and their longitudinal increase correlated with log p-tau/Aβ42 [142].

#### Role of HPA-Axis Hyperactivation

A study with 146 participants with subjective/mild cognitive impairment documented an association of EDS with reduced awakening serum cortisol level and a reduced AM/PM cortisol ratio [143]. Low awakening serum cortisol, which possibly reflects an earlier secretion than expected during the night due to e.g. increased fragmentation or arousals, was also related to reduced “feeling of recovery” after sleep [143, 144].

#### Role of Genetics

A study with 363 healthy participants (22.1 ± 2.7 years of age) showed a positive correlation between AD polygenic risk scores and higher habitual daytime sleepiness and larger SWS rebound following sleep deprivation [145]. Despite the relatively small sample size, these results suggest a role of EDS in modulating AD risk.

## Risk of AD in Sleep-Related Movement Disorders

Restless legs syndrome (RLS) is the most common sleep-related movement disorder, with a mean prevalence of 7.1%. RLS is characterised by an urge to move the legs, typically accompanied by unpleasant sensation, which emerge or intensify during rest, are partially or completely alleviated by movement, and exhibit a circadian pattern with worsening in the evening or at night. Patients with RLS may present nighttime disturbances, e.g. difficulty in initiating and continuing sleep, increased arousals with or without periodic limb movements, and daytime disturbances including affected mood, concentration and memory deficits [146]. In line with this, a recent meta-analysis found a negative association of RLS with global cognition and attention [147]. In a population-based cohort, worse language task performance were reported in patients with RLS compared to controls [148]. The dementia risk in RLS has been recently evaluated in a population study with 2,501 newly diagnosed RLS patients and 9,977 matched controls [149]. This study showed a 1.4 times increased risk of all-cause dementia, 1.8-fold risk of vascular dementia and 1.3-fold risk of AD in RLS, independent from use of dopaminergic therapy [149]. This increased risk might be speculatively attributed to overlapping risk factors for dementia in patients with RLS, e.g. poor sleep quality, depression, anxiety, and micro-vasculopathy [149]. Other studies did not show any increased risk of dementia in RLS [76, 150]. These inconsistent results highlight the need for prospective, longitudinally designed studies taking into account confounders and assessing sleep and AD biomarkers. A recent MRI study investigated the glymphatic function in 61 patients with RLS compared to 51 healthy controls [151]. The DTI-ALPS index in patients with RLS was lower compared to healthy controls, independently from clinical characteristics [151]. However, DTI-ALPS is only a proxy of glymphatic function and these findings need to be confirmed in larger independent cohorts.

## Conclusion

Understanding sleep as a modifiable risk factor for AD opens promising avenues for early detection, prevention and early intervention. As wearable technologies and sleep-monitoring tools have become widely available, integrating sleep metrics into routine health assessments could enable early identification of individuals at risk. Future research should focus on large-scale, longitudinal studies to validate sleep-based biomarkers and explore individualized interventions targeting sleep architecture and circadian regulation. Moreover, therapeutic strategies that enhance SWS and REM sleep, as well as treatments addressing sleep disorders like insomnia and OSA, hold potential not only for symptom management but also for altering neurodegenerative trajectories. Ultimately, a precision-medicine approach that combines genetic, sleep, and biomarker profiles could relevantly improve how we predict and modulate AD risk.

## Key References

- Cavaillès C, Carrière I, Wagner M, Dartigues JF, Berr C, Dauvilliers Y, et al. Trajectories of sleep duration and timing before dementia: a 14-year follow-up study. Age Ageing. 2022;51(8).
  - *Longitudinal follow-up of older adults to determine the effect of sleep duration and timing on the development of dementia*.
- Xiong Y, Tvedt J, Åkerstedt T, Cadar D, Wang HX. Impact of sleep duration and sleep disturbances on the incidence of dementia and Alzheimer’s disease: A 10-year follow-up study. Psychiatry Res. 2024;333.
  - *Longitudinal follow-up of older adults to determine the effect of sleep duration and timing on the development of dementia*.
- Baril AA, Picard C, Labonté A, Sanchez E, Duclos C, Mohammediyan B, et al. Longer sleep duration and neuroinflammation in at-risk elderly with a parental history of Alzheimer’s disease. Sleep. 2024;47(6).
  - *Longitudinal follow-up of older adults to determine the effect of sleep duration and timing on the development of dementia*.
- Liguori C, Mercuri NB, Izzi F, Romigi A, Cordella A, Sancesario G, et al. Obstructive sleep apnea is associated with early but possibly modifiable Alzheimer’s disease biomarkers changes. Sleep. 2017 May 1;40(5).
  - *Association of OSA with risk of Alzheimer’s dementia.*
- Marchi NA, Allali G, Heinzer R. Obstructive sleep apnea, cognitive impairment, and dementia: is sleep microstructure an important feature? Sleep. 2024; 47(12).:zsae161.
  - *Association of OSA with risk of Alzheimer’s dementia.*
- Fernandes M, Mari L, Chiaravalloti A, Paoli B, Nuccetelli M, Izzi F, et al. 18 F-FDG PET, cognitive functioning, and CSF biomarkers in patients with obstructive sleep apnoea before and after continuous positive airway pressure treatment. J Neurol. 2022 Oct 1;269 [10]:5356–67.
  - *Association of OSA with risk of Alzheimer’s dementia*.

## Data Availability

No datasets were generated or analysed during the current study.
